# Inadequate neuraxial anesthesia during cesarean delivery: incidence and predictors in a large retrospective contemporary cohort

**DOI:** 10.3389/fmed.2026.1930644

**Published:** 2026-10-26

**Authors:** Orhun Demir, Omer Zuhtu Yondem, Baris Ecevit Yuksel

**Affiliations:** Department of Anesthesiology, Faculty of Medicine, Lokman Hekim University, Ankara, Türkiye

**Keywords:** cesarean delivery, inadequate neuraxial block, intraoperative pain, neuraxial anesthesia, spinal anesthesia

## Abstract

**Objective:**

Spinal anesthesia is the preferred anesthetic technique for cesarean delivery because of its favorable maternal and neonatal safety profile. Despite its widespread use, inadequate neuraxial anesthesia remains an important clinical problem that may result in intraoperative pain, supplemental pain relief, or conversion to general anesthesia. Previous studies have shown inconsistent incidence rates and unreliable predictors of failed spinal anesthesia. This study aimed to determine the incidence of inadequate spinal anesthesia during cesarean delivery and to identify patient- and procedure-related factors associated with block inadequacy.

**Methods:**

This retrospective cohort study included all adult patients who underwent cesarean delivery under planned spinal anesthesia at a tertiary university hospital between January 2020 and January 2026. Inadequate spinal anesthesia was defined as the combination of partial and total failure of spinal block. Demographic, obstetric, anesthetic, and surgical variables were evaluated. Multivariable logistic regression analysis was performed to identify independent predictors of inadequate spinal anesthesia.

**Results:**

A total of 3,382 cesarean deliveries performed under spinal anesthesia were analyzed. Adequate spinal anesthesia was achieved in 3,216 patients (95.1%). Inadequate spinal anesthesia occurred in 166 patients (4.9%), including 135 partial failures (4.0%) and 31 total failures (0.9%). Increasing age and higher educational level were associated with lower odds of inadequate spinal anesthesia, whereas postpartum sterilization and surgical adhesions were independently associated with higher odds of inadequate spinal anesthesia.

**Conclusions:**

Although spinal anesthesia provided effective surgical anesthesia in the vast majority of cesarean deliveries, inadequate neuraxial block remained clinically relevant. Both patient-related and surgical factors were associated with an increased risk of block inadequacy. Identification of high-risk patients may facilitate individualized anesthetic planning and improve maternal intraoperative experience.

## Introduction

Worldwide, cesarean section (CS) is increasingly common alongside spontaneous vaginal delivery, and neuraxial anesthesia remains the preferred method for planned cesarean surgeries due to its safety and effectiveness. However, the prevalence of inadequate spinal anesthesia during cesarean delivery varies across populations and study designs, and available data are often inconsistent or context-specific. A recent meta-analysis reported a pooled incidence of failed spinal anesthesia of 8.36% (95% CI: 6.94–9.78), highlighting that failure of neuraxial techniques is a significant clinical concern (1). Another large retrospective review of 5,361 cesarean deliveries found that spinal anesthesia failed to provide a pain-free surgery in 4.1% of cases, with conversion to general anesthesia occurring in 0.7% of surgeries and supplemental analgesia or sedation required in an additional 2.0% (2). Previous cesarean delivery, concomitant tubal ligation, lower body mass index, and longer surgery duration were identified as important predictors of failure in that cohort (2). Several other observational studies have similarly reported variable failure rates and associated factors, underscoring the multifactorial nature of spinal anesthesia inadequacy (3). Although numerous risk factors have been proposed, their relative predictive value in routine obstetric practice remains unclear.

Inadequate neuraxial anesthesia during cesarean delivery has consequences beyond the need for additional analgesia or conversion to general anesthesia. Patients may experience intraoperative pain, visceral discomfort, anxiety, and reduced satisfaction with their childbirth experience. Furthermore, unplanned conversion to general anesthesia may expose both mother and neonate to additional risks and remains one of the most concerning complications of neuraxial anesthesia for cesarean delivery. Recent systematic reviews and professional recommendations have emphasized that intraoperative pain during cesarean delivery under neuraxial anesthesia should be recognized as an important quality indicator in obstetric anesthesia practice and managed promptly when encountered (4–6) Therefore, identifying patients at increased risk of inadequate spinal anesthesia may facilitate individualized anesthetic planning, improve maternal comfort, and optimize perioperative management.

Previous studies have also reported variable causes of spinal anesthesia failure, with insufficient analgesia being the most frequently reported reason in some cohorts, including a tertiary hospital series where obstetric procedures comprised a large proportion of failures (7). In a prospective observational cohort of 275 parturients, emergency procedure, needle size, anesthetist experience, and local anesthetic volume were among the factors associated with failed spinal anesthesia, and the overall failure rate was 19.5% (8). This variability in failure rates of spinal anesthesia may be related to differences in the definition of spinal anesthesia failure. Studies defining the transition to general anesthesia as failed spinal anesthesia generally report lower failure rates, while studies that also define the need for additional analgesia or sedation as failure tend to report higher rates (9–12).

Despite the growing body of literature, considerable heterogeneity exists regarding the reported incidence and predictors of inadequate spinal anesthesia, partly because of differences in outcome definitions and study populations. Additional data from contemporary obstetric cohorts may help clarify the relative importance of patient- and procedure-related factors associated with block inadequacy. Therefore, the primary objective of this retrospective cohort study was to determine the incidence of inadequate spinal anesthesia during cesarean sections at a tertiary university hospital. The secondary objectives were to identify factors associated with inadequate spinal anesthesia and to assess their independent associations with this outcome.

## Methods

This retrospective study was conducted at Lokman Hekim University Ankara Hospital between January 1, 2020, and January 1, 2026, in accordance with the Declaration of Helsinki. Ethical approval was obtained from the Lokman Hekim University Ethics Committee, Ankara, Türkiye (Date: 25/02/2026, Approval No: 2026/2-2026039). Inclusion criteria were patients who underwent cesarean delivery under spinal anesthesia, whose patient data was available, and who had a cesarean delivery record. Exclusion criteria included patients under 18 years of age, patients undergoing epidural or combined spinal-epidural anesthesia and those who underwent caesarean surgery under planned general anesthesia. Flow chart of the patient selection is demonstrated in Figure 1.

**Figure 1:**
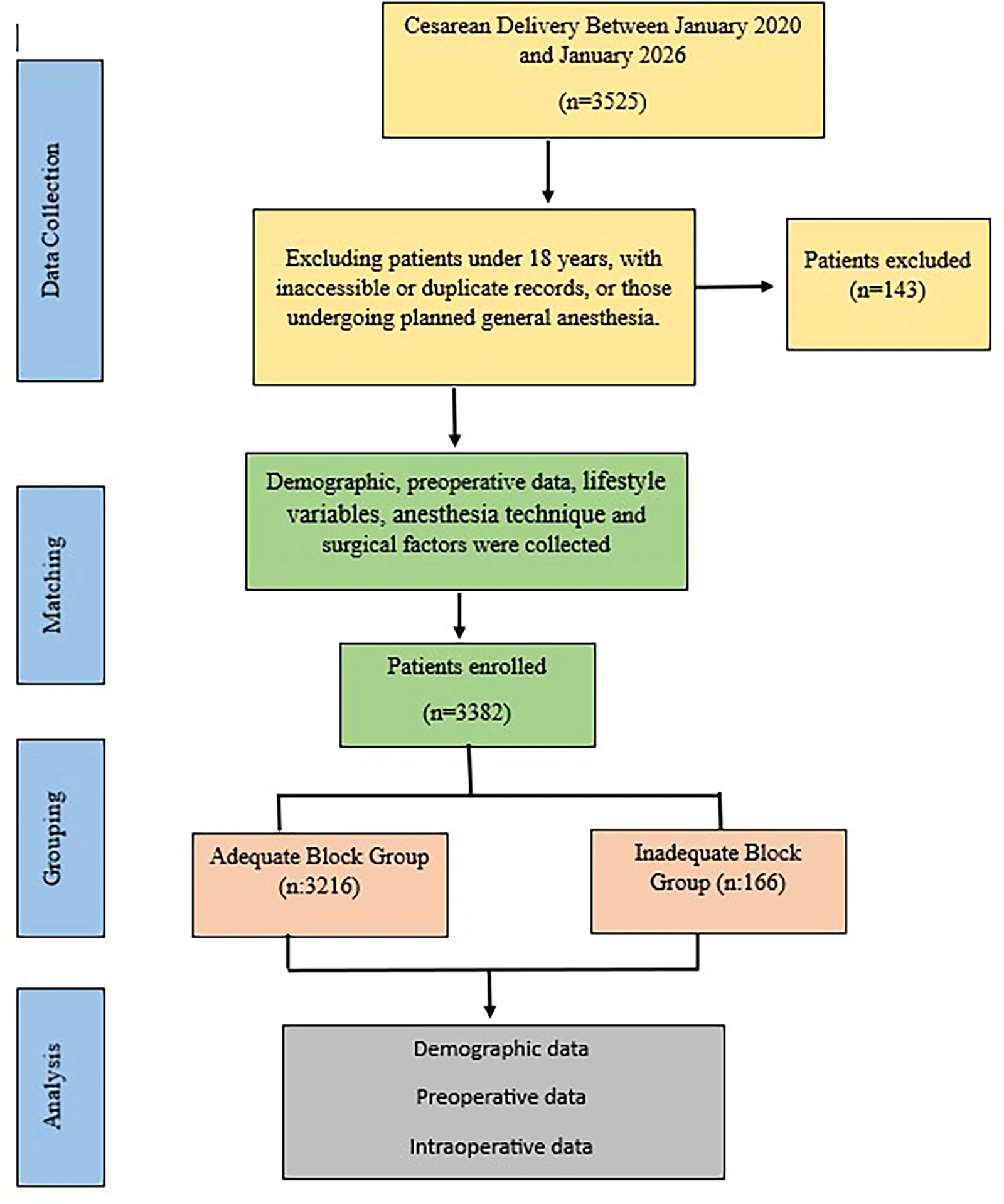
Flow diagram of patient inclusion and spinal anesthesia outcomes in cesarean delivery.

### Definitions

- Adequate spinal block: Spinal anesthesia that provided sufficient surgical anesthesia for cesarean delivery without supplemental intraoperative analgesic or sedative medication and without conversion to general anesthesia.
- Partial spinal block: this was defined as inadequate neuraxial anesthesia requiring supplemental intraoperative analgesic and/or sedative medication without conversion to general anesthesia.
- Failed spinal block: Block insufficient for surgery, requiring conversion to general anesthesia.
- Planned spinal anesthesia: Planned spinal anesthesia was defined according to the preoperative anesthesia plan documented in the electronic anesthesia record.

Patients’ data were retrospectively collected from the electronic health records of Lokman Hekim University Ankara Hospital and patient follow-up forms. Candidate variables were selected based on their clinical relevance to neuraxial anesthesia and their availability in the institutional records. Demographic and clinical variables included age, body mass index (BMI), gestational age, American Society of Anesthesiologists Physical Status (ASA-PS), and education level. Education level was categorized as primary education, high school, or university education according to the information documented in the patient records. Obstetric and surgical characteristics included postpartum sterilization and the presence of surgical adhesions. Procedural variables included patient position during spinal anesthesia and spinal needle type. These variables were extracted from the records of the index cesarean delivery.

Initially selected anesthesia technique (general or spinal anesthesia) and adequacy of neuraxial block (adequate spinal block, partial failure, or total failure) were also recorded. After standard monitoring, patients were positioned either sitting or lateral, and spinal anesthesia was performed using either Quincke or pencil-point spinal needles by experienced providers. The dose of intrathecal bupivacaine was determined according to patient characteristics, including weight and height, as part of the institutional obstetric anesthesia practice. The current guideline of the Turkish Society of Anesthesiology and Reanimation describes similar weight- and height-adjusted dosing principles (13). Cases with incomplete records were excluded before analysis.

### Statistical analysis

Patient data were analyzed using SPSS version 27 (IBM Corp., Armonk, NY, USA). Descriptive statistics were presented as median (interquartile range, IQR) for continuous variables and counts (percentages) for categorical variables. Comparisons between groups were performed using the Mann–Whitney U test for non-normally distributed continuous variables and the Chi-square test for categorical variables. Variables were selected based on clinical relevance and previously reported associations with spinal anesthesia failure. Candidate variables were considered for multivariable logistic regression based on their clinical relevance, previously reported associations, and findings from univariable analyses. Variables meeting the statistical significance criterion in univariable analyses were additionally considered for inclusion in the multivariable model. Statistical significance was set at *p* < 0.05.

## Results

A total of 3,382 cesarean deliveries performed under spinal anesthesia were included in the analysis. Adequate spinal anesthesia was achieved in 3,216 patients (95.1%). Among these patients, 166 (4.9%) experienced an inadequate spinal block, including 135 patients (4.0%) with partial failure and 31 patients (0.9%) with total failure.

Baseline demographic and clinical characteristics are presented in Table 1. Patients with inadequate spinal anesthesia were significantly younger than those with adequate block (24 [23–27] vs. 31 [28–35] years, *p* < 0.001), had higher body weight (75 [63–85] vs. 70 [62–78] kg, *p* < 0.001), and higher BMI (31.17 [27.19–35.38] vs. 27.11 [23.83–31.11] kg/m^2^, *p* < 0.001). However, height was slightly lower in the inadequate spinal anesthesia group (155 [151–157] vs. 157 [151–169] cm, *p* < 0.001). Gestational age during delivery did not differ significantly between the groups (p:0.145).

**Table 1:** Relationship between patient characteristics, procedural factors, and spinal block effectiveness.

Variables	Adequate Block (n:3216)	Inadequate Block (n:166)	*P*
Age (median, IQR, years)	31 (28–35)	24 (23–27)	˂0.001
Weight (median, IQR, kg)	70 (62–78)	75 (63–85)	˂0.001
Height (median, IQR, cm)	157 (151–169)	155 (151–157)	˂0.001
Gestational age (median, IQR, weeks)	38 (38–39)	38 (38–40)	0.145
BMI (median, IQR, kg/m^2^)	27.11 (23.83–31.11)	31.17 (27.19–35.38)	˂0.001
Education			
Primary Education *n* (%)	387 (92.6%)	31 (7.4%)	0.004
High school *n* (%)	1865 (94.8%)	102 (5.2%)	
University *n* (%)	964 (96.7%)	33 (3.3%)	
ASA PS group			
ASA PS 2 *n* (%)	2967 (95%)	155 (5%)	0.709
ASA PS 3 *n* (%)	249 (95.8%)	11 (4.2%)	
Position			
Sitting *n* (%)	3203 (95.3%)	159 (4.7%)	˂0.001
Lateral *n* (%)	13 (65%)	7 (35%)	
Needle Type			
Quincke needle *n* (%)	2243 (93.4%)	149 (6.6%)	0.277
Pencil point needle *n* (%)	979 (98.9%)	11 (1.1%)	
Postpartum sterilization			
Not Performed *n* (%)	3211 (95.8%)	142 (4.2%)	˂0.001
Performed *n* (%)	5 (17.2%)	24 (82.8%)	
Surgical adhesions			
Not Observed *n* (%)	3205 (95.2%)	161 (4.8%)	˂0.001
Observed *n* (%)	11 (68.8%)	5 (31.32%)	

IQR: Interquartile Range; BMI: Body Mass Index; ASA PS: American Society of Anesthesiologists Physical Status.

Percentages for categorical variables are presented within each category.

Among categorical variables, education level was significantly associated with spinal block adequacy, higher education degree was associated with lower incidence of inadequate block. Rate of inadequate spinal block among patients graduated from university and primary school were 3.3% and 7.4% respectively. ASA PS classification showed no significant association with block adequacy. Regarding surgical variables, additional postpartum sterilization and the presence of previous surgical adhesions were both found to be significantly associated with inadequate spinal anesthesia. Namely, among patients who did not undergo postpartum sterilization, 142 of 3,353 (4.2%) experienced an inadequate spinal block, whereas among those who underwent postpartum sterilization, 24 of 29 (82.8%) experienced an inadequate spinal block. Patients with observed surgical adhesions had a higher rate of inadequate spinal block than those without adhesions (5/16, 31.3% vs. 161/3,366, 4.8%, respectively; *p* < 0.001). An adequate spinal block was achieved in 3,203 of 3,362 patients (95.3%) in the sitting position, compared with 13 of 20 patients (65.0%) in the lateral position (*p* < 0.001).

Variables, ASA PS and type of needle were not found to be associated with adequacy of spinal anesthesia with *p* values of 0.709 and 0.277 respectively.

We conducted logistic regression analysis to identify factors associated with inadequate spinal block. In univariate analysis, increasing age was associated with lower odds of inadequate spinal block (OR: 0.642, 95% CI 0.605–0.681; *p* < 0.001), whereas higher BMI (OR: 1.148, 95% CI 1.119–1.179; *p* < 0.001), lateral position (OR: 10.847, 95% CI 4.269–27.562; *p* < 0.001), postpartum sterilization (OR: 108.40, 95% CI 42.50–276.50; *p* < 0.001), and the presence of surgical adhesions (OR: 9.05, 95% CI 3.12–26.24; *p* < 0.001) were associated with higher odds of inadequate spinal block. Higher education level was also associated with lower odds of inadequate spinal block (OR: 0.653, 95% CI 0.509–0.837; *p* < 0.001).

In multivariable logistic regression analysis, increasing age remained associated with lower odds of inadequate spinal block (OR: 0.524, 95% CI 0.479–0.573; *p* < 0.001). Postpartum sterilization (OR: 9.535, 95% CI 2.750–33.056; *p* < 0.001) and the presence of surgical adhesions (OR: 3.010, 95% CI 1.120–8.760; *p* = 0.020) were independently associated with higher odds of inadequate spinal block, whereas higher education level was associated with lower odds (OR: 0.678, 95% CI 0.495–0.928; *p* = 0.015). BMI and lateral position were not independently associated with inadequate spinal block after adjustment for potential confounding factors.

The results of the logistic regression analyses are presented in Table 2.

**Table 2:** Univariate and multivariable logistic regression analysis of factors associated with inadequate spinal block.

Predictors	Univariate regression analysis			Multivariate regression analysis		
	OR	95% CI	p	OR	95% CI	p
Age (years)	0.642	0.605–0.681	˂0.001	0.524	0.479–0.573	˂0.001
BMI (kg/m^2^)	1.148	1.119–1.179	˂0.001	0.634	0.366–1.096	0.103
Lateral Positions	10.847	4.269–27.562	˂0.001	1.514	0.115–2.291	0.383
Postpartum sterilization	108.40	42.50–276.50	˂0.001	9.535	2.750–33.056	˂0.001
Surgical adhesions	9.05	3.12–26.24	˂0.001	3.010	1.120–8.760	0.020
Education levels	0.653	0.509–0.837	˂0.001	0.678	0.495–0.928	0.015

OR: Odds Ratio; CI: Confidence Interval; BMI: Body Mass Index

## Discussion

In this large contemporary cohort, inadequate spinal anesthesia occurred in 4.9% of cesarean deliveries, with most cases representing partial rather than complete block failure. Increasing age and higher educational level were associated with lower odds of inadequate block, whereas postpartum sterilization and surgical adhesions were independently associated with higher odds after multivariable adjustment.

Younger patients may experience higher levels of perioperative anxiety, greater pain sensitivity, or lower tolerance to visceral discomfort during surgical manipulation. Such factors may influence the perception of anesthetic adequacy and increase the likelihood that supplemental sedation or analgesia is required during cesarean delivery (5, 6). In our cohort, younger maternal age emerged as an independent predictor of inadequate spinal anesthesia as well. Although this association has not been consistently reported across studies, several patient-related factors may contribute to this finding. The strong inverse association between maternal age and inadequate neuraxial anesthesia observed in this cohort should be interpreted cautiously. Although variable coding was rechecked, additional model-diagnostic analyses were not available to establish the robustness of this estimate. Further studies are needed to determine whether this association is reproducible in other obstetric populations.

Education level also emerged as an independent predictor in our study. Although this factor has rarely been evaluated in previous research, differences in patient expectations, communication and perception of intraoperative sensations may influence the reporting of discomfort during neuraxial anesthesia. Patients with higher educational levels may have a clearer understanding of perioperative explanations and intraoperative sensations, which may influence how they interpret and report intraoperative discomfort (2, 6, 14).

Higher BMI was associated with inadequate spinal anesthesia in the univariate analysis but did not remain an independent predictor after multivariable adjustment. The relationship between obesity and outcomes of neuraxial anesthesia remains complex. From a physiological perspective, obesity may theoretically enhance the cephalad spread of intrathecal local anesthetics through increased intra-abdominal pressure and reduced cerebrospinal fluid volume (10, 15). However, technical challenges frequently encountered in patients with increased body habitus may counterbalance these theoretical advantages. Identification of anatomical landmarks may be more difficult, the depth to the intrathecal space may be greater, and needle placement may be less precise (10, 15, 16). These opposing physiological and technical mechanisms may explain the inconsistent association between BMI and neuraxial block adequacy reported across previous studies and may account for the loss of significance after adjustment for potential confounding factors in our cohort (1). The associations of BMI and patient position with inadequate neuraxial anesthesia differed between univariable and multivariable analyses. These changes may reflect adjustment for other covariates; however, the available analyses do not allow the contribution of individual factors or potential model instability to be determined. Accordingly, the adjusted estimates should be interpreted cautiously.

In addition to patient characteristics, several surgical variables were associated with inadequate spinal anesthesia. Postpartum sterilization was identified as an independent predictor in our cohort. Tubal ligation performed during cesarean delivery often requires additional traction on the fallopian tubes and increased peritoneal manipulation, which may provoke visceral discomfort even in the presence of an otherwise adequate somatic block (4, 6). As a result, these procedures may increase the likelihood that supplemental sedation or analgesia is required. Postpartum sterilization has also been identified as a significant predictor of inadequate spinal anesthesia in obstetric cohorts, with some studies reporting a strong association between additional surgical manipulation and the need for supplemental analgesia or sedation during cesarean delivery under neuraxial block (2). Separate analyses examining neuraxial anesthesia for postpartum tubal ligation have reported relatively high failure rates even with *de novo* spinal techniques, further supporting the idea that additional surgical procedures may challenge the adequacy of single-shot spinal anesthesia (17).

The presence of surgical adhesions was another factor independently associated with inadequate spinal anesthesia in our study. Adhesions may increase surgical complexity, prolong operative duration, and require more extensive visceral manipulation. Under these circumstances, the duration or intensity of surgical stimulation may exceed the analgesic coverage provided by a single-shot spinal technique (17). Prior observational studies have also noted that increased surgical manipulation, including exteriorization or complications during obstetric surgery, can be associated with greater requirements for supplemental analgesia, suggesting that surgical factors beyond patient characteristics may influence neuraxial block adequacy (18).

A large prospective cohort study evaluating spinal anesthesia failure during cesarean delivery identified several procedural and provider-related risk factors, including less experienced providers (< 1 year), patient positioning (particularly lateral), multiple puncture attempts, presence of bloody cerebrospinal fluid, obesity, and lower intrathecal bupivacaine dose (19). Patient positioning was not independently associated with spinal anesthesia failure in our cohort. These findings likely reflect the standardized spinal anesthesia protocols and the experience of anesthesia providers at our institution. A summary of previous studies on spinal anesthesia failure during cesarean delivery is presented in Table 3.

**Table 3:** Literature review on spinal anesthesia failure during cesarean delivery.

Author, year	size	Design	Country	Population	Total Failure, *n* (%)	Inadequate Block, *n* (%)
Jin et al. 2024	5361	Retrospective	China	Obstetrics	37 (0.7%)	112 (2.1%)
Ahmad et al. 2024	149	Prospective	Pakistan	Obstetrics	4 (2.7%)	11 (8.7%)
Demilie et al. 2024	532	Prospective	Ethiopia	Obstetrics and non-obstetrics	6 (5%)	119 (22.4%)
Punchuklang et al. 2022	12001	Retrospective	Thailand	Obstetrics	100 (0.8%)	110 (0.9%)
Yüksek et al. 2020	1004	Retrospective	Türkiye	Obstetrics and non-obstetrics	167 (16.6%)	NA
At et al. 2011	414	Prospective	Nigeria	Obstetrics	25 (6.0%)	14 (3.4%)
Bekele et al. 2022	794	Prospective	Ethiopia	Obstetrics	35 (4.4%)	86 (10.8%)
Ashagrie et al. 2019	275	Prospective	Ethiopia	Obstetrics	3 (1.09%)	54 (19.5%)
Bagle et al. 2024	3933	Prospective	India	Obstetrics and non-obstetrics	62 (1.57%)	72 (1.83%)
Current Study	3382	Retrospective	Türkiye	Obstetrics	31 (0.9%).	166 (4.9%)

NA: Not Applicaple

Spinal anesthesia provides highly effective surgical anesthesia for cesarean delivery, although incomplete blockade remains a clinically relevant limitation of single-shot spinal techniques. In this large contemporary cohort, inadequate spinal block occurred in 4.9% of cases and was associated with specific patient- and procedure-related factors. Certain patient and surgical characteristics may increase the likelihood of inadequate neuraxial blockade requiring supplemental intraoperative management. Early recognition and management of inadequate neuraxial anesthesia are also emphasized in current recommendations addressing intraoperative pain during cesarean delivery (14).

### Limitations

Several limitations should be considered when interpreting our findings. First, the retrospective design limits control over potential confounding and relies on the accuracy of medical record documentation. Second, some procedural variables, including the number of puncture attempts, emergency status, operative duration, and intrathecal anesthetic dose or volume, were unavailable or inconsistently documented and therefore could not be evaluated. Consequently, residual confounding cannot be excluded, particularly for the associations of postpartum sterilization and surgical adhesions with inadequate spinal anesthesia. Third, the exact indications for supplemental intraoperative analgesic or sedative medications could not be reliably determined from the available records. Finally, differences in the definitions of spinal anesthesia failure across studies may limit direct comparisons with previous cohorts.

## Conclusion

In this large retrospective cohort, spinal anesthesia provided effective surgical anesthesia for cesarean delivery, although incomplete blockade remains a clinically relevant limitation of single-shot spinal techniques. Certain patient- and procedure-related characteristics were associated with an increased likelihood of inadequate neuraxial blockade requiring supplemental intraoperative management. Identification of these factors has important implications for obstetric anesthesia practice. Preoperative recognition of patients at increased risk may allow anesthesiologists to optimize anesthetic planning and facilitate individualized management to ensure adequate maternal comfort during cesarean delivery.

## Main points

1. Clinically relevant inadequate neuraxial anesthesia occurred in 4.9% of 3,382 cesarean deliveries, whereas conversion to general anesthesia was required in only 0.9% of patients, indicating that clinically important block inadequacy extends beyond complete spinal failure.
2. Increasing age and higher educational level were associated with lower odds of inadequate neuraxial anesthesia, whereas postpartum sterilization and surgical adhesions were independently associated with higher odds of inadequate neuraxial anesthesia after multivariable adjustment.
3. Most inadequate neuraxial blocks were managed with supplemental intraoperative analgesia or sedation rather than conversion to general anesthesia, emphasizing the clinical importance of recognizing partial block failure.
4. Reliance on conversion to general anesthesia alone may underestimate the true burden of inadequate neuraxial anesthesia and supports a broader, clinically relevant definition of block inadequacy in obstetric anesthesia research.

## Data Availability

The datasets presented in this article are not readily available because the datasets generated and/or analyzed during the current study are not publicly available because they contain confidential patient information and are subject to institutional and ethical restrictions. De-identified data may be made available by the corresponding author upon reasonable request and with permission from the Lokman Hekim University Ethics Committee, in accordance with institutional policies and applicable regulations. Requests to access the datasets should be directed to orhun.demir@lokmanhekim.edu.tr.
